# Anti-inflammatory effect of SGLT-2 inhibitors via uric acid and insulin

**DOI:** 10.1007/s00018-022-04289-z

**Published:** 2022-05-03

**Authors:** Rosalba La Grotta, Paola de Candia, Fabiola Olivieri, Giulia Matacchione, Angelica Giuliani, Maria Rita Rippo, Elena Tagliabue, Monica Mancino, Francesca Rispoli, Sabina Ferroni, Cesare Celeste Berra, Antonio Ceriello, Francesco Prattichizzo

**Affiliations:** 1 IRCCS MultiMedica, PST, Via Fantoli 16/15, 20138 Milan, Italy; 2 Department of Molecular Medicine and Medical Biotechnology, University of Naples “Federico II”, Naples, Italy; 3 Department of Clinical and Molecular Sciences, DISCLIMO, Università Politecnica Delle Marche, Ancona, Italy; 4 Center of Clinical Pathology and Innovative Therapy, IRCCS INRCA, Ancona, Italy; 5 Value-Based Healthcare Unit, IRCCS MultiMedica, Sesto San Giovanni, MI Italy; 6 Laboratory Medicine, IRCCS MultiMedica, Milan, Italy; 7 Department of Endocrinology, Nutrition and Metabolic Diseases, IRCCS MultiMedica, Via Milanese 300, 20099 Sesto San Giovanni, MI Italy

**Keywords:** Sodium–glucose cotransporter 2 inhibitors, IL-6, Uric acid, Insulin, Ketones, Low-grade inflammation, Diabetes complications, Cardiovascular diseases, Kidney disease

## Abstract

Sodium–glucose cotransporter 2 (SGLT-2) inhibitors (i) reduce cardiovascular and renal events in patients with and without type 2 diabetes (T2D). However, the underlying mechanisms are debated. Low-grade inflammation (LGI) is a key driver of vascular complications, suggested to be attenuated by SGLT-2i in animal models. Based on a specific working hypothesis, here we investigated the net effect of SGLT-2i on LGI in patients with T2D and the possible underlying mechanism. We enrolled patients with T2D treated either with a stable therapy with SGLT-2i or with other glucose-lowering drugs (GLD) (*n* = 43 per group after matching for a range of pro-inflammatory variables), and tested hs-CRP and interleukin (IL)-6 as primary variables of interest. Patients treated with SGLT-2i had lower circulating levels of IL-6, a prototypical marker of LGI, but also of uric acid and fasting insulin, compared with patients treated with other GLD. Then, to explore whether uric acid and insulin might mediate the effect of SGLT-2i on IL-6, we tested physiologically pertinent doses of these two molecules (i.e. 0.5 mM uric acid and 1 nM insulin) in two in vitro models of LGI, i.e. monocytes (THP-1) treated with LPS and endothelial cells (HUVEC) exposed to hyperglycaemia. Results from in vitro models supported a pro-inflammatory role for uric acid and its combination with insulin in monocytes and for uric acid alone in hyperglycaemia-stimulated endothelial cells. On the contrary, we observed no drug-intrinsic, anti-inflammatory effect for dapagliflozin, empagliflozin, and canagliflozin in the same models. Overall, these results suggest that SGLT-2i possess a tangible activity against LGI, an effect possibly mediated by their ability to lower uric acid and insulin concentrations and that juxtaposes other proposed mechanisms in explaining the observed benefit of this class on cardiovascular and renal endpoints.

**Supplementary Information:**

The online version contains supplementary material available at 10.1007/s00018-022-04289-z.

## Introduction

Sodium–glucose cotransporters (SGLTs) are integral membrane proteins that operate the transport of glucose across membranes. While SGLT-1 are expressed also in the small intestine and mediate glucose absorption [1], SGLT-2 are almost exclusively expressed in the kidney [2], and in particular in the early proximal tubule where they promote the reabsorption of 80–90% of the glucose filtered by the glomerulus [3].

SGLT-2 inhibitors (i) are a class of glucose-lowering drugs (GLD) promoting glucose elimination through urine [4]. Multiple trials and large observational studies have demonstrated a marked protective effect of SGLT-2i against major adverse cardiovascular (CV) events, hospitalizations for heart failure, CV-related and all-cause death, and renal outcomes [5–8]. Of note, SGLT-2i have also been observed to improve heart failure- and kidney-related endpoints in patients without type 2 diabetes (T2D) [9, 10]. In addition, the observation that other GLD (oGLD) show similar potency in improving glycaemic control without improving CV nor other outcomes suggests that the benefit of SGLT-2i does not depend only on their ability to lower glycaemia, as also confirmed by mediation analyses [11, 12].

Understanding the underpinnings of the beneficial effect of SGLT-2i might be of relevance: (1) to identify patients that benefit most from their use; (2) to encourage further drug designs or drug repurposing with specific beneficial effects; and (3) to provide a rationale for an extended use in patients without diabetes [9, 10]. Several hypotheses have been posed to explain such beneficial effects, encompassing a large range of intermediate risk factors, e.g. blood pressure, sodium balance, body weight, metabolic switches (e.g. ketones increase), haemodynamic adjustments, hormonal alterations, endothelial function, uric acid, sympathetic nervous system activity, cardiac sodium–hydrogen exchangers, cardiac reverse remodelling, anti-oxidant and anti-inflammatory pathways [13–16], the latter substantiated by experimental evidence in animal models [17, 18]. While fewer data are available for humans, two studies using the same population of patients showed a decrease of selected inflammatory mediators in subjects treated with SGLT-2i compared to those treated with sulphonylureas and reaching the glycaemic equipoise [19, 20]. However, no details regarding the underlying mechanisms were explored [19, 20], nor were these findings reproduced using a comparator population on a therapy different from sulphonylureas.

Low-grade inflammation (LGI) is an increasingly recognised driver of CV diseases and other vascular complications, especially in patients with T2D [21–23]. Among the large range of soluble factors characterising LGI, hs-CRP and IL-6 have been suggested to predict future CV and renal events in patients with T2D [23–27]. In particular, hs-CRP has been repeatedly reported to be an independent risk factor for CV events [28] and CV mortality [29]. Similarly, IL-6 levels improve CV risk stratification on top of canonical risk factors in patients with T2D [30]. Thus, IL-6 and especially hs-CRP might be considered two “sensors” of LGI with a prognostic value for the development of diabetes complications. We have previously proposed a conceptual framework where SGLT-2i are hypothesised to promote an increase in ketone bodies, such as β-hydroxybutyrate (BHB), and a decrease in the circulating levels of uric acid and insulin to cumulatively ameliorate LGI, independently of canonical risk factors, e.g. HbA1c and LDL cholesterol [17].

To test this hypothesis, we recruited patients with T2D on stable therapy with SGLT-2i and compared them with patients on oGLD, matched for a range of canonical risk factors known to affect LGI. We set hs-CRP and IL-6 as the primary variables of interest, while white blood cell (WBC) count, myeloperoxidase (MPO) concentration, hs-troponin I, BHB, insulin, and uric acid were exploratory variables. Then, to explore the mechanism-related results obtained in this cross-sectional cohort, we studied the effect of physiologically pertinent doses of uric acid, insulin, and their combination in two well-established in vitro models of diabetes-related LGI, i.e. lipopolysaccharide (LPS)-induced inflammation in monocytes and hyperglycaemia-stimulated endothelial cells [31, 32].

## Materials and methods

### Sample size calculations

Given its role in sensing LGI and in predicting CV complications [24], hs-CRP was selected as the parameter to compute the needed sample size. Considering a hypothetical mean level of 1.8 ± 0.5 mg/L in one group and assuming a difference of up to 1.5 ± 0.3 mg/L in patients on another drug, as observed in other studies [33], a sample size of 41 subjects for each group reaches 90% power to reject the null hypothesis of equal means between patients treated with SGLT-2i and patients on oGLD, considering that the difference between the means of the two populations is *μ*1–*μ*2 = 1.8–1.5 = 0.3 with a standard deviation of 0.5 for the first group and 0.3 for the second group, with alpha = 5% and using a two-way *t*-test tails for two samples with unequal variance. Such sample size was considered adequate also to detect differences in the mean levels of IL-6. Indeed, one study found a mean difference of 0.5 pg/ml (confidence interval − 0.2 to −0.7) in the mean levels of IL-6 when comparing patients on SGLT-2i with patients on sulphonylureas [20]. The formulas provided by the Cochrane handbook [34] were used to calculate the standard deviation and then an online statistical calculator [35] was used to estimate the needed sample size by entering the difference between two means and the expected standard deviation, which yielded a required sample size of 43 for each group to achieve a power of 80% and a level of significance of 5% (two sided).

### Study design and patients’ characteristics

One hundred patients were initially recruited in the study, 52 on SGLT-2i and 48 on oGLD (Supplementary Fig. 1). Through the use of the electronic health record of the people attending the Diabetology unit of IRCCS MultiMedica, patients with T2D, older than 18 years, with the ability and willingness to give written informed consent and on a stable therapy from at least 6 months, i.e. no new prescription of any drug, were recruited. Exclusion criteria were diabetes different from T2D, pregnancies, short life expectancy, chronic or acute inflammatory conditions, i.e. cancer, autoimmune diseases, and hs-CRP > 10 mg/L, a commonly used cutoff underlying an acute or chronic pro-inflammatory condition [36]. Information relative to anthropometric factors, medical history, therapies, the presence/absence of diabetes complications were extracted from the electronic health record. Two blood samples (one from each group) were haemolyzed and thus excluded from the analysis. Three patients in the SGLT-2i group and two in the oGLD group had hs-CRP levels > 10 mg/L and were eliminated from the analysis. To allow for matching of the necessary characteristics, five patients in the SGLT-2i group and two in the oGLD group were also excluded, thus yielding a final cohort of 43 patients per group (Supplementary Fig. 1), matched for age, sex, diabetes duration, BMI, fasting glycaemia, HbA1c, total cholesterol, LDL cholesterol, triglycerides, prevalence of hypertension, statin use, prevalence of diabetes complications, background use of other GLD and CV drugs (Table 1).

**Table 1:** Clinical characteristics of the cohort

Variable	SGLT-2i (*n* = 43)	oGLD (*n* = 43)	*p*-value
Age (years)	64.3 (56.8–71)	65.6 (59.8–72)	0.441
Gender (males, %)	22 (51.2%)	23 (53.4%)	0.903
Diabetes duration (years)	10.6 ± 4.9	11.04 ± 7.1	0.716
BMI (kg/m^2^)	29.5 ± 4.8	29.9 ± 4.6	0.700
Waist circumference (cm)	105.3 ± 10.8	102.3 ± 8	0.489
Total cholesterol (mg/dL)	165.7 (137.8–183.3)	176.8 (153.8–197.8)	0.075
LDL-C (mg/dL)	93.1 ± 30.2	100.4 ± 27.1	0.212
HDL-C (mg/dL)	46.2 (39–52)	50.6 (40.5–55.8)	0.258
Triglycerides (mg/dL)	132.5 (94.5–156.3)	129.3 (97.8–147.5)	0.904
Fasting glucose (mg/dL)	128 ± 23	130.8 ± 21	0.751
HbA1C (%)	6.6 (6.0–6.9)	6.6 (6.2–6.9)	0.810
Creatinine (mg/dL)	0.9 (0.74–1.11)	0.92 (0.7–0.99)	0.865
eGFR (ml/min per 1.73 m^2^)	81.4 (70.8–92.1)	82.6 (80.9–95.4)	0.453
Hypertension (*N*, %)	30 (69.8%)	21 (48.8%)	0.317
Systolic blood pressure (mmHg)	121.9 ± 10.3	118.3 ± 9.8	0.438
Diastolic blood pressure (mmHg)	73.5 ± 7.1	70.8 ± 8	0.415
Smokers (*N*, %)	6 (13.9%)	7 (16.3%)	0.796
Macrovascular complications (*N*, %)	12 (27.9%)	10 (23.3%)	0.704
Microvascular complications (*N*, %)	13 (30.2%)	19 (44.2%)	0.365
Background GLD (*N*, %)			
Metformin	40 (93%)	40 (93%)	1
Sulphonylureas	1 (2.3%)	2 (4.7%)	0.570
Glitazones	5 (11.6%)	3 (7%)	0.498
GLP-1RA	5 (11.6%)	10 (23.2%)	0.233
DPP-4i	1 (2.3%)	5 (11.6%)	0.114
Insulin	11 (25.6%)	5 (11.6%)	0.168
Other CV drugs (*N*, %)			
Statins	26 (60.5%)	23 (53.5%)	0.732
Other lipid-lowering drugs	5 (11.6%)	5 (11.6%)	1
ACE inhibitor/ARB (*N*, %)	15 (34.9%)	19 (44.2%)	0.561
Calcium channel blockers (*N*, %)	4 (9.3%)	4 (9.3%)	1
Beta blockers (*N*, %)	11 (25.6%)	6 (13.9%)	0.267
Diuretics (*N*, %)	6 (13.9%)	6 (13.9%)	1
Aspirin	11 (25.6%)	10 (23.2%)	0.845
Other antiplatelet or anticoagulant drugs	5 (11.6%)	6 (13.9%)	0.776

Continuous variables are described as mean ± SD for parametric variables and as median (interquartile range) for non-parametric variables. Categorical variables are reported as number (*N*) with the relative percentage (%). The relative *p*-values derive from Mann–Whitney *U* test for continuous non-parametric variables, Student’s *t*-test for continuous parametric variables, and Chi-squared test for categorical variables

*BMI* body mass index, *LDL-C* low-density lipoprotein cholesterol, *HDL-C* high-density lipoprotein cholesterol, *HbA1c* glycated haemoglobin, *eGFR* estimated glomerular filtration rate (through CKD-EPI), *GLP-1RA* glucagon-like peptide 1 receptor agonists, *DPP-4i* dipeptidyl-peptidase 4 inhibitor

The primary variables of interest were hs-CRP and IL-6. WBC, MPO, hs-troponin I, BHB, insulin, and uric acid were exploratory variables. Patients on therapy with insulin were excluded by the comparison of fasting insulin levels.

The study protocol was in accordance with the Declaration of Helsinki and was approved by the local ethics committee (IRCCS MultiMedica, Milan, Italy, protocol n.MM335.2018). All participants provided their written informed consent.

### Laboratory measurements and ELISAs

Blood samples were collected after overnight fasting. Fasting glycaemia, HbA1c, total cholesterol, LDL cholesterol, triglycerides, hs-CRP, WBC, troponin, insulin, and uric acid were measured by standard procedures. One EDTA blood tube was centrifuged at 2000*g* for 15 min at 4 °C to obtain EDTA plasma samples, immediately stored at −80 °C. Plasma EDTA samples were then used to measure IL-6 (BMS213HS, Invitrogen), MPO (BMS2038INST, Invitrogen), and BHB (MAK041, Sigma) with ELISA (IL-6 and MPO) or colorimetric (BHB) kits, according to manufacturer’s instructions.

### Cell cultures and reagents

Human monocytic THP-1 cells (ATCC) were maintained in RPMI-1640 medium supplemented with 10% heat-inactivated foetal bovine serum, 1% penicillin/streptomycin, and 1% l-glutamine (all from Euroclone). The cells were seeded at a density of 2 × 10^5^ cells/ml in T75 flasks (Eppendorf).

Human umbilical vein endothelial cells (HUVEC) from pooled donors (Clonetics, Lonza) were cultured in endothelial basal medium (EBM-2, CC-3156, Lonza) supplemented with SingleQuot Bullet Kit (CC-4176, Lonza). The cells were seeded at a density of 5000/cm^2^ in T75 flasks.

For the experiments, 1 × 10^6^ cells/ml THP-1 were pre-treated with 0.5 mM uric acid (U2625, Sigma), 1 nM insulin (I-034, Sigma), or their combination for 3 h. Then, 0.1 μg/ml LPS (L6529, Sigma) was added for additional 4 h. The same concentrations of uric acid and insulin were used to treat 8000/cm^2^ HUVEC together with 25 mM glucose (G8270, Sigma) for 1 week. Control cells were untreated and not exposed to osmotic equipoise. The dose of uric acid and insulin were selected based on previous literature [37, 38] and are in line with the physiological range observed in patients. In another set of experiments, 2 μM dapagliflozin (SML2804, Sigma), 5 μM canagliflozin (S2760, Selleckchem), and 2 μM empagliflozin (S8022, Selleckchem) were used either to pre-treat THP-1 for 3 h before a 4-h stimulation with LPS or as a co-treatment in HUVEC exposed to 25 mM glucose. At the end of the experiments, the cells were counted and the conditioned media were centrifuged to remove debris and collected.

### RNA extraction and RT qPCR for mRNA expression

Total RNA was extracted with the RNA purification kit (Norgen Biotek) and checked for concentration and purity with Nanodrop (Thermo Fisher). Sample with a 260/280 ratio of ~ 2.0 were selected and 1 μg of RNA was reverse-transcribed with Superscript III RT kit (Invitrogen) according to the manufacturer’s instructions. Real-time PCR (RT-PCR) was performed in a QuantStudio 6 Flex (Applied Biosystems) detection system using SybrGreen reagents (Takara Bio Company). For normalisation purposes, 18S was used as the reference gene [39]. The thermal profile used was as previously published [32, 40]. The list of primers used can be found in Supplementary Table 1.

### Western blot and IL-1β measurement

Cells were lysed in RIPA buffer with 10% protease inhibitor (11873580001 cOmplete™, EDTA-free Protease Inhibitor Cocktail, Roche). Protein concentration was determined using the Bradford assay (B6916, Sigma). For Western blot analysis, 50 µg of lysate was separated by electrophoresis using PAGE gels (NuPAGE 4–12% Bis–Tris Gel, Thermo Fisher) and transferred to PVDF membranes (Amersham Hybond P 0.45 PVDF 10600023). After blocking with 5% non-fat dried milk, membranes were incubated overnight at 4 °C with the following primary antibodies: anti-IL-8 (ab110727; Abcam, dilution 1:1000), anti-IL-6 (#12153; Cell Signalling, 1:1000), and anti-β-actin (#8457S; Cell Signalling, 1:1000). Secondary IgG HP-conjugated anti-rabbit HRP-linked antibody (#7074; Cell Signalling, 1:3000) were applied for 1 h at room temperature. Immunoreactive proteins were revealed with SuperSignal™ West Pico (ThermoFisher) using UVITEC Alliance Q9. β-Actin was used as the loading control. Densitometric analysis was performed with Image J software. To measure released IL-1β, conditioned medium was collected as described above and IL-1β was measured through ELISA (BMS224-2, Invitrogen), according to manufacturer’s instructions. Cell number was used to normalise cytokine concentration.

### Statistical analysis

Variables were tested for normality using the Shapiro Wilk’s test. Continuous variables were reported as mean ± SD for parametric variables and as median (interquartile range) for non-parametric variables. To compare the characteristics in the two groups of the cohort, Mann–Whitney *U* test was used for non-parametric variables, two-sample Student’s *t*-test was used for parametric variables and the Chi-squared test was used for categorical variables. Given the non-normal distribution of the variables of interest, Mann–Whitney *U* test was used to compare the levels of such parameters in the two groups. The prevalence of patients with hs-CRP > 2 mg/L in the two groups was compared with the Chi-squared test. Spearman’s coefficient was used to estimate the correlation between IL-6 and either uric acid or insulin. For in vitro experiments, the one-way ANOVA followed by Tukey–Kramer test was used to compare the effect of the different treatments. The analyses were carried out using Prism 7 (GraphPad). Statistical significance was defined as a *p*-value of < 0.05.

## Results

### Lower levels of circulating IL-6, uric acid and insulin in patients on SGLT-2i

**Fig. 1:**
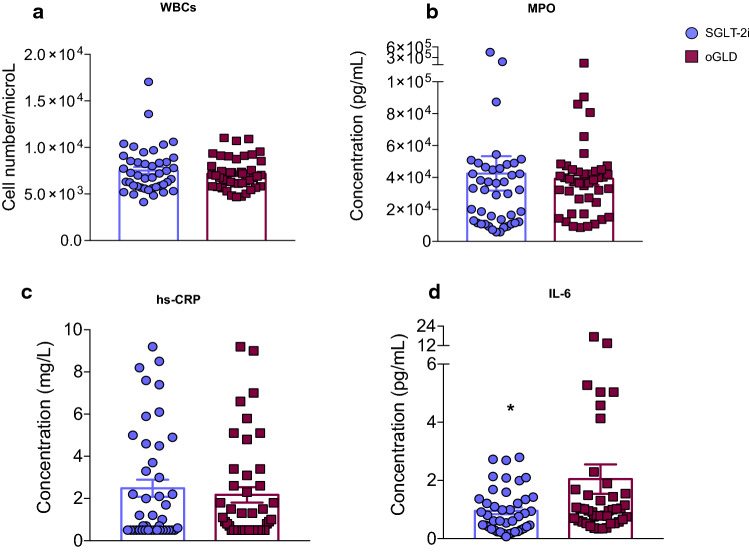
SGLT-2i users have lower levels of IL-6. Circulating levels of IL-6 (**A**), hs-CRP (**B**), MPO (**C**), and the number of white blood cells (WBC) (**D**) in patients on therapy with SGLT-2i (blue circles) and patients on oGLD (red squares). Data are presented as mean ± SD. *n* = 43 per group. *p < 0.05 Mann–Whitney *U* test. *IL-6* interleukin 6, *hs-CRP* high-sensitivity C-reactive protein, *MPO* myeloperoxidase, *oGLD* other glucose-lowering drugs

The group of patients treated with SGLT-2i (*n* = 43) and that of patients on oGLD (*n* = 43), matched as described above (Table 1), did not show differences in blood circulating WBC count (Fig. 1A) nor in MPO concentration (Fig. 1B). Similarly, the analysis of immune cells populations revealed no quantitative differences between the two groups (Supplementary Fig. 2). The levels of hs-CRP were also not significantly different between the two groups (Fig. 1C). Since having hs-CRP levels > 2 mg/L has been suggested as a risk factor for CV diseases [41], we compared the prevalence of this condition in the two groups and found no difference in the percentage of patients with this risk factor in the two groups (Supplementary Fig. 3). However, patients of the SGLT-2i group showed significantly lower circulating levels of IL-6 when compared with the oGLD group (Fig. 1D).

**Fig. 2:**
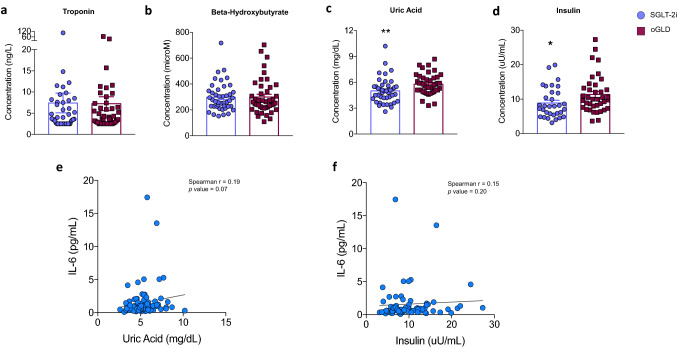
SGLT-2i users have lower levels of insulin and uric acid. Circulating levels of troponin (**A**), β-hydroxybutyrate (**B**), insulin (**C**), and uric acid (**D**) in patients on therapy with SGLT-2i (blue circles) and patients on oGLD (red squares). Spearman’s correlations between uric acid and IL-6 (**E**), and between insulin and IL-6 (**F**). Data are presented as mean ± SD. *n* = 43 per group for panel **A**, **B**, and **D**. *n* = 32 in the SGLT-2i group and *n* = 38 in the oGLD group for panel **C**. *n* = 86 for panel **E**. *n* = 70 for panel **F**. *p < 0.05 Mann–Whitney *U* test. *IL-6* interleukin 6, *oGLD* other glucose-lowering drugs

Relative to the exploratory variables, the two groups showed no differences in terms of circulating levels of hs-troponin I (Fig. 2A) and BHB (Fig. 2B). On the contrary, uric acid (Fig. 2C) and insulin levels (Fig. 2D) were significantly lower in the SGLT-2i group compared with the oGLD group. To gain preliminary insights about the possible associations among either uric acid or insulin and IL-6, we explored a possible correlation between such variables [42]. Spearman’s coefficients suggested a certain degree of correlation between either uric acid (Fig. 2E) or insulin (Fig. 2F) and IL-6.

### Role of uric acid, insulin, and their combination in LPS-induced inflammation in monocytes

**Fig. 3:**
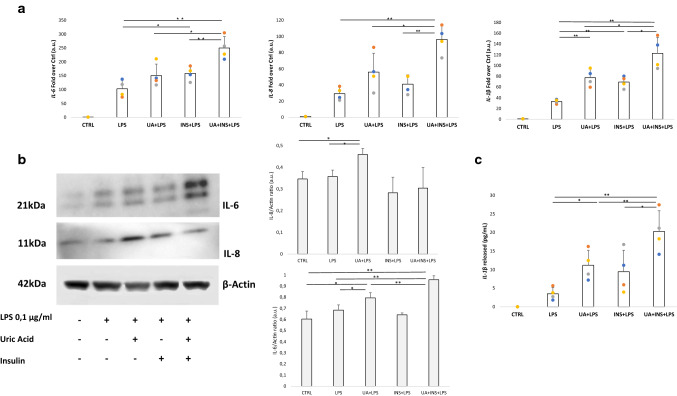
Uric acid and insulin promote LPS-induced inflammation in monocytes. Expression of *IL-6, IL-8,* and *IL-1β* mRNA (**A**), IL-6 and IL-8 proteins along with the relative densitometry (**B**), and the amount of released IL-1β (**C**) in THP-1 exposed to LPS, uric acid, insulin, and their combination. Data are presented as mean ± SD. *n* = 4. *p < 0.05; **p < 0.01 one-way ANOVA followed by Tukey–Kramer test

To explore whether uric acid, insulin, and their combination can boost inflammatory responses in monocytes, we exposed THP-1 cells to 0.5 mM uric acid, 1 nM insulin, or their combination for 3 h, before stimulating cells with 0.1 μg/ml LPS for an additional 4 h. The combination of uric acid and insulin significantly increased mRNA expression of three inflammatory mediators, *IL-6*, *IL-8* and *IL-1β* when compared with either LPS alone or LPS + uric acid, which itself potentiated the response induced by LPS (Fig. 3A). These results were substantiated by Western blot experiments showing an increase in the expression of IL-6 and IL-8 proteins in cells exposed to uric acid, with IL-6 being further augmented by the combination of uric acid and insulin (Fig. 3B). The levels of IL-1β released in culture medium reflected the same trend (Fig. 3C), suggesting that uric acid, and more strongly the uric acid-insulin combination, can boost the inflammatory response induced by LPS in monocytes, increasing the production of IL-6.

### Role of uric acid, insulin, and their combination in hyperglycaemia-induced inflammation in endothelial cells

**Fig. 4:**
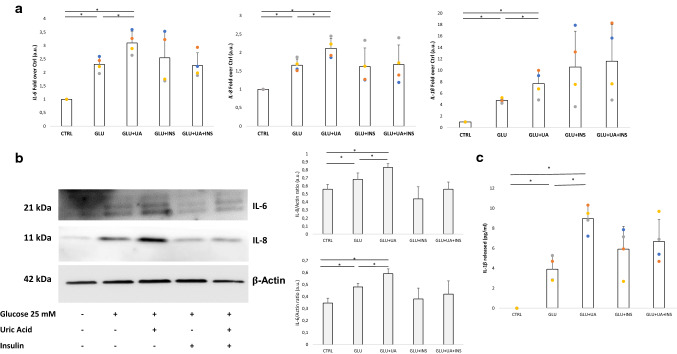
Uric acid, but not insulin, potentiates hyperglycaemia-induced inflammation in endothelial cells. Expression of *IL-6, IL-8,* and *IL-1β* mRNA (**A**) and IL-6 and IL-8 proteins along with the relative densitometry (**B**), and the amount of released IL-1β (**C**) in HUVEC exposed to hyperglycaemia, uric acid, insulin, and their combination for 1 week. Data are presented as mean ± SD. *n* = 4. *p < 0.05 one-way ANOVA followed by Tukey–Kramer test

To assess whether uric acid, insulin, and their combination can foster inflammatory responses in endothelial cells exposed to hyperglycaemia, we treated HUVEC with 25 mM glucose for 1 week, with the same concentrations of uric acid, insulin, and their combination. Uric acid significantly increased the expression of *IL-6, IL-8* and *IL-1β* mRNAs when compared with hyperglycaemia alone, which itself induced a significant pro-inflammatory response (Fig. 4A). These results were corroborated by Western blot showing an increase in the expression of IL-6 and IL-8 proteins in cells exposed to hyperglycaemia, with uric acid further inducing their expression (Fig. 4B). On the contrary, insulin, either alone or in combination, did not modulate the expression of any of these mediators (Fig. 4A and B). The same pattern was observed when testing the levels of IL-1β released in the culture media (Fig. 3C).

### Absence of intrinsic anti-inflammatory properties for common SGLT-2i

Since some reports advanced the hypothesis that specific SGLT-2i possess intrinsic anti-inflammatory properties [43, 44], we tested the three most commonly prescribed SGLT-2i, i.e. dapagliflozin, canagliflozin, and empagliflozin [45] in the same in vitro models, using the maximum concentration achievable by these drugs in plasma of treated patients [46–48]. None of the SGLT-2i tested was able to hamper the increase in the mRNA expression of *IL-6*, *IL-8* and *IL-1β* induced by LPS in THP-1 or by hyperglycaemia in HUVEC (Supplementary Fig. 4), suggesting that these drugs do not have intrinsic anti-inflammatory properties in these settings.

## Discussion

The use of SGLT-2i is being increasingly expanded to prevent the progression of heart failure and chronic kidney disease, also in patients without T2D [49]. Despite this success, the mechanisms explaining such benefit are still matter of investigation. The majority of mechanistic studies has focussed on a range of haemodynamic effects, especially considering the rapidly-emerging benefit in terms of heart failure and kidney-related endpoints [13–16]. However, SGLT-2i reduce the incidence of a larger range of endpoints, e.g. atherosclerosis-related outcomes and all-cause mortality, as shown in both clinical trials and large observational studies [8, 49].

**Fig. 5:**
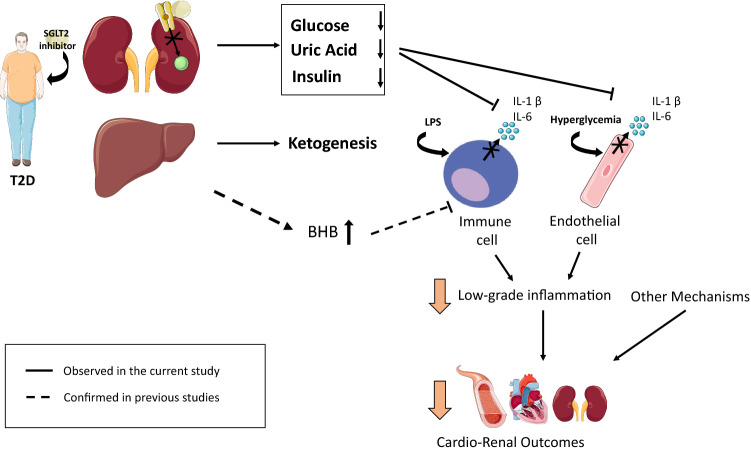
Schematic representation of the proposed mechanisms underlying the effect of SGLT-2i against low-grade inflammation. SGLT2 inhibitors promote the elimination of glucose through the kidneys, an effect accompanied by an increased excretion of uric acid, a decrease in insulin release, and an increase in mean levels of ketone bodies such as β-hydroxybutyrate (BHB). Immune cells secrete inflammatory cytokines including IL-1β and IL-6 in response to hyperglycaemia, insulin and uric acid concentrations, while BHB inhibits this process [37 and this paper]. Uric acid also potentiates the inflammatory response induced by hyperglycaemia in endothelial cells [this paper]. Thus, SGLT-2i might ameliorate systemic LGI through multiple mechanisms. This could eventually impact the development of diabetes complications, especially in the long term

LGI is a pervasive feature of T2D, underlying the development of virtually the whole spectrum of diabetes complications [21–27]. We previously hypothesised that SGLT-2i might attenuate chronic LGI, given their ability to increase circulating levels of ketones and to reduce insulin and uric acid levels, three mechanisms converging on a reduced LGI [17]. Here, we tested this hypothesis by enrolling patients on stable therapy with SGLT-2i and comparing them to patients on oGLD but matched for a range of known pro-inflammatory variables. We showed that patients on therapy with SGLT-2i have lower circulating levels of IL-6, a prototypical marker of LGI with a recognised prognostic value for the development of diabetes complications [26, 27]. In our setting, this effect might be mediated by the lower levels of uric acid and insulin observed in patients with in the SGLT-2i group, rather than by ketone elevation, since we detected no difference in the circulating levels of fasting BHB. On the other side, previous work has largely substantiated the increase of BHB after treatment with SGLT-2i, when considering both the fasting and the fed state [50]. This discrepancy might be attributable to our choice of enrolling patients on stable therapy from at least 6 months, which eventually have influenced the results. Indeed, published metabolic studies with SGLT-2i usually last few weeks [49]. Of note, a recent manuscript clearly demonstrated an anti-inflammatory effect linked to the increase of BHB after initiation of SGLT-2i [37]. Indeed, while our study was being conducted, another group tested a similar hypothesis by harvesting macrophages from SGLT-2i treated patients and compared them to the same cells isolated from patients treated with sulphonylureas to reach the glycaemic equipoise after one month of therapy. They showed a reduction in IL-1β secretion in the SGLT-2i group compared to sulphonylureas, an effect accompanied and mediated by the increase in BHB and the decrease in insulin levels [37]. Our results extend these data to a larger range of cytokines and to a wider temporal window. Indeed, while we did not detect a difference in BHB levels, we were still able to observe lower levels of IL-6 together with decreased circulating insulin and uric acid, with functional experiments supporting a potential pro-inflammatory role for these two mediators. A reconciling framework considering all these observations might thus suggest that SGLT-2i produce an initial acute anti-inflammatory effect lowering IL-1β secretion through the activity of BHB on the inflammasome, while longer-term administration of SGLT-2i complement this action by constantly promoting lower levels of insulin and uric acid, which in turn result in decreased levels of IL-6, overall ameliorating LGI (Fig. 5).

Uric acid is a well-established pro-inflammatory mediator [38]. On the contrary, the effect of insulin on LGI is more debated, with different reports suggesting a context, dose, and time-dependent activity [51, 52]. However, a recent manuscript clearly showed a pro-inflammatory role for insulin in peritoneal macrophages, likely a result of a mechanism needed to optimise acute glycaemic control [53]. Indeed, insulin increases the uptake of glucose into macrophages, thus reinforcing a pro-inflammatory pattern via the insulin receptor, glucose metabolism, and the production of reactive oxygen species. Of note, postprandial inflammation was prevented by SGLT-2i, which attenuated both hyperglycaemia and insulin release [53]. These data are in accordance with our results showing that physiologically pertinent doses of insulin further potentiate the inflammatory response induced by uric acid in monocytes. On the other side, a pro-inflammatory effect of insulin was not observed in endothelial cells exposed to hyperglycaemia, in line with previous observations [52].

The lack of a direct anti-inflammatory effect for three commonly prescribed SGLT-2i observed in our models does not exclude the possibility that such drugs might hold an intrinsic anti-inflammatory activity in other cell types or in case of different inflammatory stimuli [43]. For instance, in another study, canagliflozin inhibited the pro-inflammatory effect induced by IL-1β in endothelial cells [43]. In addition, given our results, we did not explore the expression of SGLT-2 in endothelial cells nor in monocytes. Previous manuscripts have shown that both HUVEC and aortic endothelial cells express this transporter [43], while no data are available for THP-1. Moreover, other studies evidenced that the expression of SGLT-2 in non-renal tissues might be induced by specific triggers [54], thus suggesting that a putative, direct effect of SGLT-2i might be context-dependent.

A relevant question is whether having lower levels of IL-6 is clinically meaningful. In the CANTOS trial, an antibody inhibiting the action of IL-1β was able to reduce the incidence of CV events in a population largely composed by patients with T2D or prediabetes [55]. Of note, in the group of patients achieving on-treatment IL-6 < 1.65 ng/L, the burden of total CV events was considerably lower compared with those above this median level [56]. Thus, considering that the mean level of IL-6 in the SGLT-2i group falls below this cut-off while that of oGLD group stands above the same threshold, the results presented here are likely to be of clinical relevance. Similarly, another study suggested that IL-6 levels ≥ 3.3 pg/ml are a powerful predictor of long-term CV mortality in patients in secondary CV prevention [57]. Of note, our results suggest that seven patients in the oGLD group but none of the SGLT-2i group had IL-6 levels above this threshold. In addition, our results were observed in a cohort with a highly prevalent use of metformin and statins, drugs known to ameliorate LGI [58, 59], and with well-controlled risk factors. Indeed, both groups had the mean or median level of multiple risk factors, e.g. HbA1c and lipids, within the targets suggested by current guidelines [60] (Table 1), thus corroborating the potential net anti-inflammatory effect of SGLT-2i.

Beyond CV diseases, microvascular complications are also known to be promoted by LGI and in particular by IL-6 [22, 27]. In this respect, two recent publications using the same cohort showed that patients treated with SGLT-2i have lower levels of IL-6 [19, 20], with a mechanism of action network model identifying IL-6 as a SGLT-2i-sensitive, central hub in the development and progression of diabetic nephropathy [19]. The relevance of targeting this cytokine in patients with diabetic nephropathy is further stressed by an ongoing trial testing an anti-IL-6 biological, i.e. ziltivekimab, in patients with chronic kidney disease and with high hs-CRP levels, with the majority of patients having diabetic kidney disease [61]. Since blocking IL-6 efficiently reduced LGI, the investigators will test whether such approach is able to reduce the burden of CV events in this at-risk population [61]. Of note, the populations enrolled in the CV outcomes trials showing a reduction in CV events in patients treated with SGLT-2i were similar to that of the abovementioned trial, since they were at high risk for atherosclerosis or in secondary CV prevention [11]. In addition, SGLT-2i seems to be particularly effective against CV events in patients with moderate or severe renal impairment [9, 62].

To our knowledge, hs-CRP and IL-6 were not explored as possible mediators of the beneficial effect of SGLT-2i in the CV outcomes trials, likely due to the limited possibility of assessing many variables in such large populations [11, 17]. The same applies to insulin levels. On the contrary, uric acid was suggested to mediate part of the beneficial effect of empagliflozin on CV and kidney-related endpoints in the EMPA-REG trial [12]. Beyond the academic interest, identifying the mediators of the benefit of SGLT-2i could be of utmost importance for at least two reasons: (1) to provide mechanistic bases to extend the use of these drugs; (2) to identify patients who would benefit the most by the use of SGLT-2i [11, 15, 63]. Relatively to this latter point, Sen and colleagues have recently explored whether three other mediators of LGI, namely tumour necrosis factor receptor (TNFR)-1, TNFR-2 and kidney injury molecule-1 (KIM-1), acted as intermediate risk factors of the renal benefit provided by canagliflozin in the CANVAS trial [64]. They showed that early decrease in TNFR-1 and TNFR-2 during canagliflozin treatment was independently associated with a lower risk of kidney disease progression, suggesting that markers of LGI have the potential to be pharmacodynamic markers of response to SGLT-2i [64].

A limitation of our study is its cross-sectional nature, which inherently impedes to firmly establish that the effect on IL-6 is only ascribable to SGLT-2i. However, given the very stringent design of the study, we considered the risk for residual confounders to be limited. On the other hand, we did not record the dietary habits nor the amount of physical activity performed by the patients, which might have influenced the results. In addition, the comparator drugs were not a specific class, thus we could not perform a possible head-to-head comparison with drugs known to ameliorate LGI such as metformin [67]. In addition, the study was adequately powered to detect differences in hs-CRP and IL-6, but eventually not for uric acid and insulin, as well as to perform correlations. On the other hand, the effect of SGLT-2i on uric acid and insulin levels is very well documented by multiple studies [65, 66]. Thus, the results presented here have unlikely been obtained by chance, albeit larger numbers are needed to adequately explore correlations among variables.

In summary, we have here showed that patients on therapy with SGLT-2i are characterised by an attenuated LGI, as shown by lower levels of IL-6, an effect possibly mediated by decreased levels of uric acid and insulin. LGI accompanies a large range of Western diseases beyond T2D [15, 67]. If the activity of SGLT-2i against LGI is confirmed, the use of such drugs might be extended to a large range of conditions, also beyond heart failure and kidney disease [9, 10]. In addition, these results reinforce the postulate that LGI is a tangible and eventually druggable phenomenon in T2D [15, 67]. In addition, since no marker tested so far identifies the patients benefitting most of the treatment [68], these and other results [64] might suggest that patients with the highest degree of LGI can eventually profit more than others in terms of reduction of hard endpoints, a hypothesis deserving further exploration. However, the anti-inflammatory mechanism proposed here likely juxtaposes other pathways and phenomena in mediating the beneficial effect of SGLT-2i on cardiorenal and other endpoints. Our results suggest that LGI might represent an intermediate risk factor that should be further studied to eventually explore which patients can benefit most by the use of SGLT-2i and whether such drugs can be employed to treat other conditions characterised by a pervasive LGI status.

## Supplementary Information

Below is the link to the electronic supplementary material.

Supplementary file1 (PDF 350 kb)

## Data Availability

All data generated or analysed during this study are included in this published article and supplementary information files.
