# Supplementary material for: Anti-inflammatory effect of SGLT-2 inhibitors via uric acid and insulin
Source: Cell Mol Life Sci. 2022 May 3;79(5):273. doi: 10.1007/s00018-022-04289-z (PMC9064844; doi:10.1007/s00018-022-04289-z)
Supplement: Supplementary file 1 — Supplementary file1 (PDF 350 kb) [file 18_2022_4289_MOESM1_ESM.pdf]

Supplementary Figure 1. Flow chart of the study.

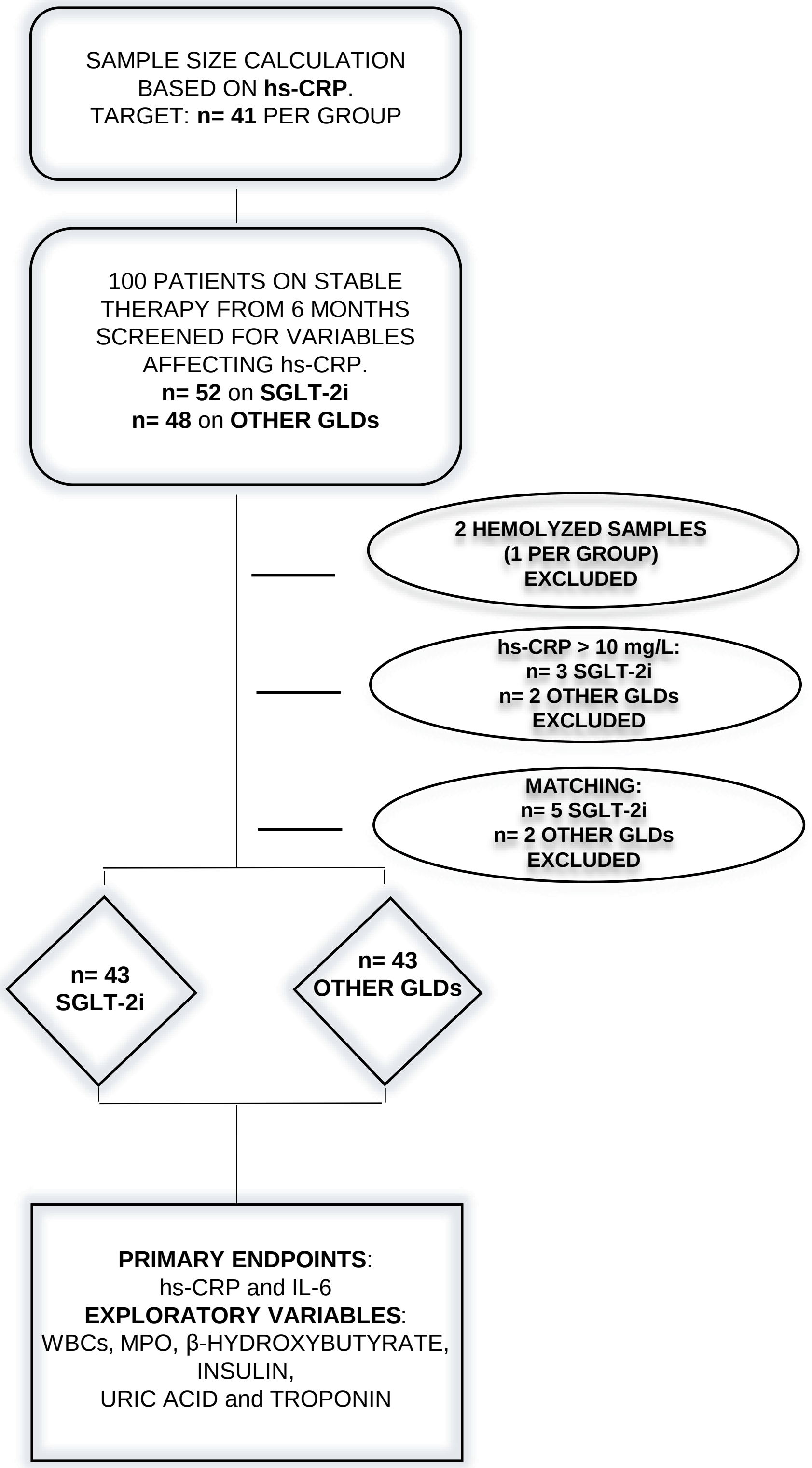

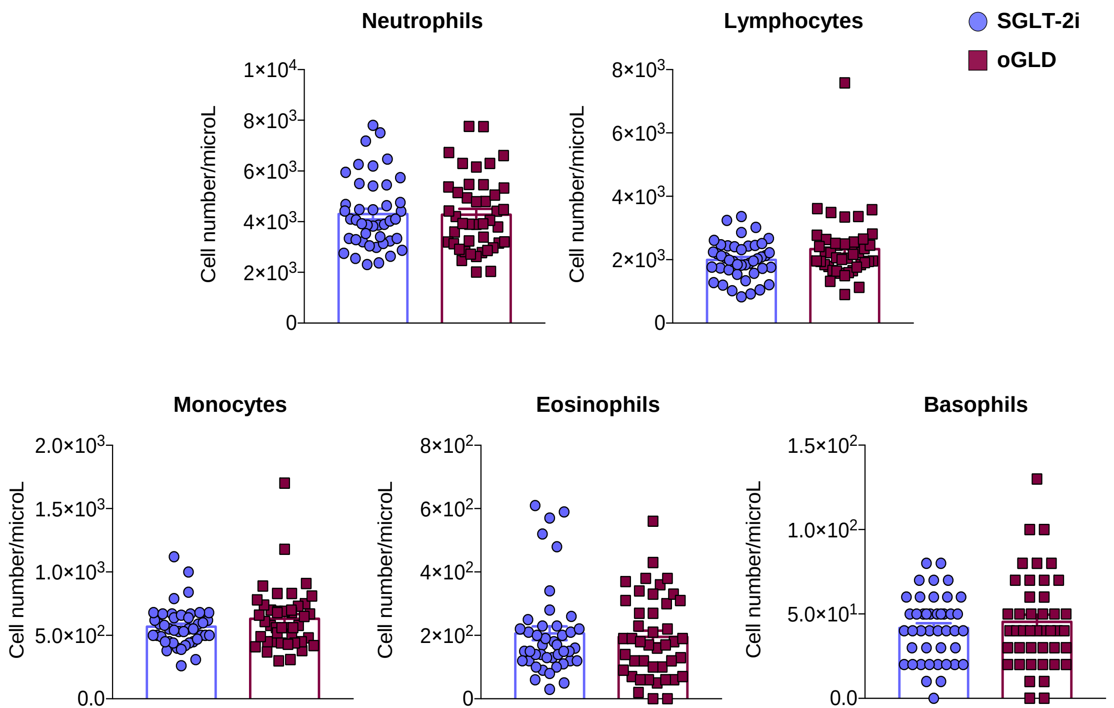

Supplementary Figure 2. Immune cell populations. Amount of neutrophils, lymphocytes, monocytes, eosinophils, and basophils in the groups of patients treated with SGLT-2i (blue) or with oGLD (red). No significant difference was observed (Mann-Whitney U test).

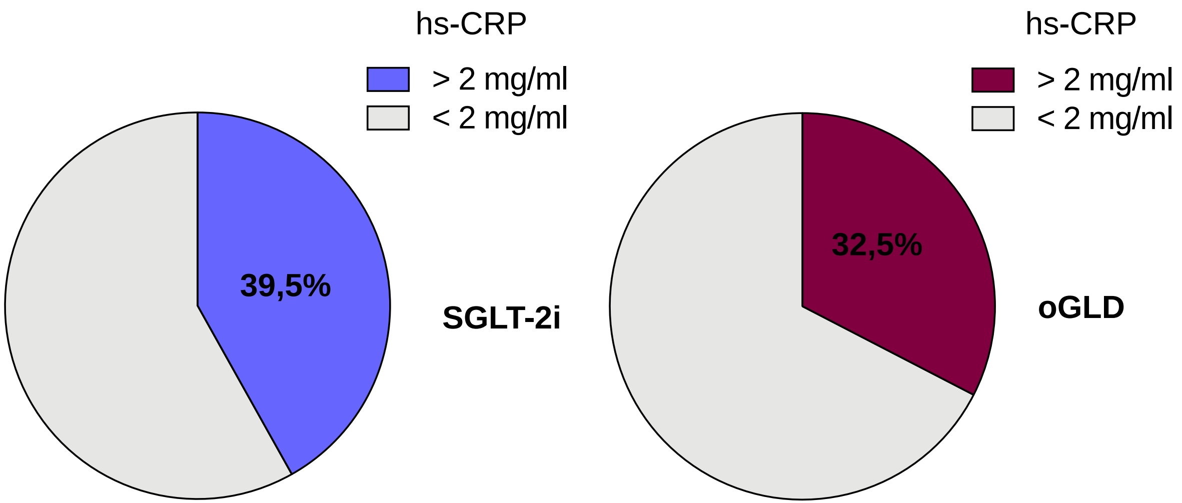

Supplementary Figure 3. Proportion of patients with residual inflammatory risk, *i.e.* with hs-CRP > 2mg/ml in the groups of patients treated with SGLT-2i (blue) or with oGLD (red). No significant difference was observed (chi squared test).

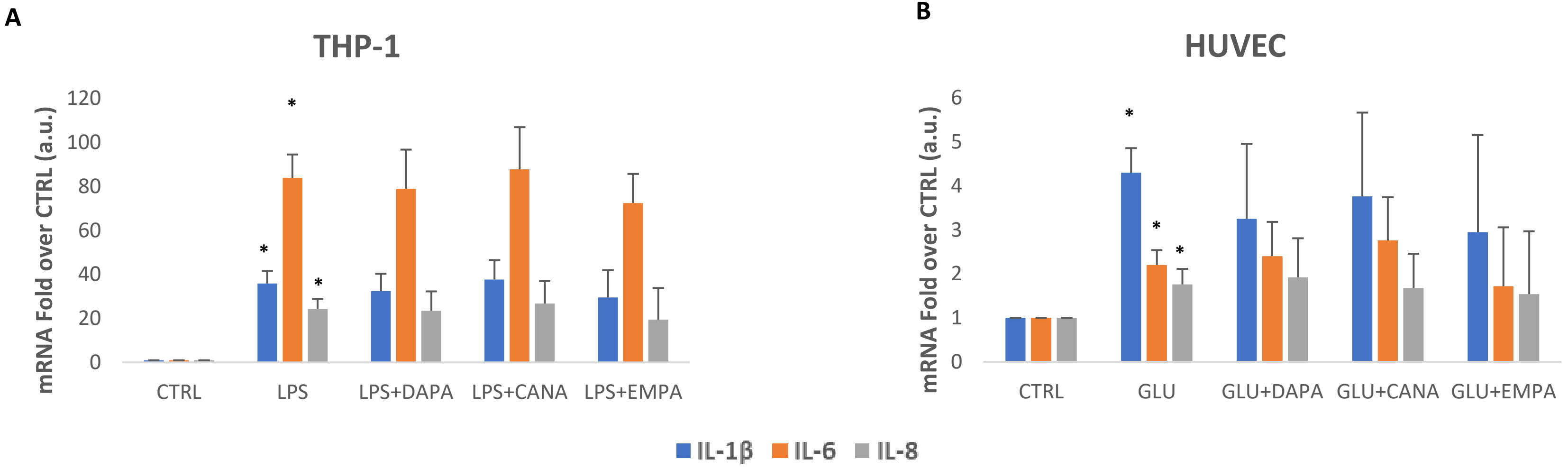

Supplementary Figure 4. SGLT-2i do not have intrinsic anti-inflammatory properties. mRNA expression of *IL-1β, IL-6,* and *IL-8* in THP-1 monocytes treated with 0,1 µg/ml LPS for 4 hours (**A**) or endothelial cells (HUVEC) treated with 25mM glucose for one week (**B**). Dapagliflozin 2uM (DAPA), canagliflozin 5uM (CANA), or empagliflozin 2uM (EMPA) were added as co-treatments before LPS stimulation for 3 hours (A) or continuously during the whole experiment (B). Data are presented as mean ± SD. n= 3. * p < 0,05 vs CTRL. One-way ANOVA followed by Tukey-Kramer test.

Supplementary Table 1. List of the primers' sequences used in the study.

**Primers**	**Sequence**
IL-6 For	TGC AAT AAC CAC CCC TGA CC
IL-6 Rev	GTG CCC ATG CTA CAT TTG CC
IL-8 For	GGA CAA GAG CCA GGA AGA AA
IL-8 Rev	CCT ACA ACA GAC CCA CAC AAT A
IL-1 Beta For	GGA CAA GCT GAG GAA GAT GC
IL-1 Beta Rev	TCG TTA TCC CAT GTG TCG AA
18S For	CGC AGC TAG GAA TAA TGG AAT AGG
18S Rev	CAT GGC CTC AGT TCC GAA A
